# Predictors of Epstein-Barr virus serostatus and implications for vaccine policy: A systematic review of the literature

**DOI:** 10.7189/jogh.10.010404

**Published:** 2020-06

**Authors:** Joanne R Winter, Charlotte Jackson, Joanna EA Lewis, Graham S Taylor, Olivia G Thomas, Helen R Stagg

**Affiliations:** 1 Centre for Molecular Epidemiology and Translational Research, Institute for Global Health, University College London, London, UK; 2 MRC Clinical Trials Unit, University College London, London, UK; 3 National Institute for Health Research (NIHR) Health Protection Research Unit in Modelling Methodology and Medical Research Council Centre for Outbreak Analysis and Public Health, Department of Infectious Disease Epidemiology, Imperial College London, London, UK; 4 Institute of Immunology and Immunotherapy, College of Medical and Dental Sciences, University of Birmingham, Birmingham, UK; 5 Usher Institute, University of Edinburgh, Edinburgh, UK; * Contributed equally and listed alphabetically.

## Abstract

**Background:**

Epstein-Barr virus (EBV) is an important human pathogen; it infects >90% people globally and is linked to infectious mononucleosis and several types of cancer. Vaccines against EBV are in development. In this study we present the first systematic review of the literature on risk factors for EBV infection, and discuss how they differ between settings, in order to improve our understanding of EBV epidemiology and aid the design of effective vaccination strategies.

**Methods:**

MEDLINE, Embase, and Web of Science were searched on 6^th^ March 2017 for observational studies of risk factors for EBV infection. Studies were excluded if they were published before 2008 to ensure relevance to the modern day, given the importance of influencing future vaccination policies. There were no language restrictions. After title, abstract and full text screening, followed by checking the reference lists of included studies to identify further studies, data were extracted into standardised spreadsheets and quality assessed. A narrative synthesis was undertaken.

**Results:**

Seventy-seven papers met our inclusion criteria, including data from 31 countries. There was consistent evidence that EBV seroprevalence was associated with age, increasing throughout childhood and adolescence and remaining constant thereafter. EBV was generally acquired at younger ages in Asia than Europe/North America. There was also compelling evidence for an association between cytomegalovirus infection and EBV. Additional factors associated with EBV seroprevalence, albeit with less consistent evidence, included ethnicity, socioeconomic status, other chronic viral infections, and genetic variants of HLA and immune response genes.

**Conclusions:**

Our study is the first systematic review to draw together the global literature on the risk factors for EBV infection and includes an evaluation of the quality of the published evidence. Across the literature, the factors examined are diverse. In Asia, early vaccination of infants would be required to prevent EBV infection. In contrast, in Western countries a vaccine could be deployed later, particularly if it has only a short duration of protection and the intention was to protect against infectious mononucleosis. There is a lack of high-quality data on the prevalence and age of EBV infection outside of Europe, North America and South-East Asia, which are essential for informing effective vaccination policies in these settings.

Epstein-Barr Virus (EBV) is a herpesvirus that infects 90%-95% of humans, causing lifelong infection [1,2]. EBV infection during childhood is generally asymptomatic, however acquisition of EBV during adolescence or early adulthood often causes infectious mononucleosis (IM) [3]. EBV is associated with 1% of global cancers, particularly Hodgkin’s lymphoma, Burkitt’s lymphoma, nasopharyngeal cancer (NPC) and gastric cancer [4].

EBV infection is not currently treatable or preventable by vaccination, although the National Institutes of Health have stated that an EBV vaccine is an important goal [5]. Vaccines are currently in development which could protect against EBV infection or EBV-associated disease [6]. It is critical for vaccine development to know the target product profile for a potential vaccine, which is only possible with a good understanding of the underlying epidemiology of infection and disease.

The epidemiology of EBV infection is not well understood. Seroprevalence increases with age, and seropositivity appears to occur at younger ages in resource-limited countries [7,8]. Whilst 90%-95% of people are infected by age 25, a small proportion (5%-10%) remain seronegative throughout their life [9]. EBV infection is correlated with human cytomegalovirus (CMV) infection [10,11], another herpesvirus which infects a high proportion of the population from a young age and is linked to reduced life expectancy [12]. Unlike CMV, the impact of EBV seronegativity on the life course is unknown. Our immune systems and EBV are likely well-adapted to each other, given the co-evolution of humans and virus [13,14]. Beyond this, studies of EBV epidemiology are sparse, and there has been no attempt to synthesise the global literature on the factors associated with EBV serostatus. Such syntheses are critical, as a greater understanding of EBV epidemiology, including the dynamics of EBV infection in different sub-populations and the long-term health impacts of remaining EBV-seronegative, will be necessary for the vaccine development.

Given the importance of systematically collating, describing and formal critical evaluations of the evidence, we conducted the first systematic epidemiological literature review of risk factors for EBV infection, and discuss the implications of our findings for future EBV vaccination policy.

## METHODS

### Search strategy and study selection

MEDLINE, Embase, and Web of Science were searched on 6th March 2017 for articles on risk factors for EBV infection. The search terms included variations of ‘human herpesvirus 4’, ‘Epstein-Barr virus’, ‘infectious mononucleosis’, ‘glandular fever’, ‘serostatus’, ‘risk factor’, ‘cross-sectional study’, ‘cohort study’, ‘case-control study’, ‘clinical trial’ and ‘human’; the full search terms are shown in Supplementary item 1. Studies published before 2008 were excluded to ensure that the risk factors reported are relevant in the present day. Title, abstract and full-text screening was split between HRS and CJ, with 10% overlap. Disagreements were resolved by consensus. Studies were included if they contained data on the percentage of people with antibodies against EBV stratified by age or any other risk factor. Studies were excluded if they examined risk factors for EBV-associated disease (eg, IM, cancers or autoimmune conditions) rather than infection, if there was no comparison group, or if they did not include original research (reviews, editorial pieces, case reports).

### Data extraction, synthesis and quality assessment

Data extraction and quality assessment was split between HRS and JRW, with 10% overlap to ensure consistency. Data were extracted into a pre-designed spreadsheet that recorded the study design, study population, and EBV seroprevalence, stratified by risk factor. Discrepancies between reviewers were resolved by consensus. Studies published in languages other than English were extracted by an additional reviewer, with additional quality control by JRW. Where studies reported data on risk factors for multiple EBV antigens, we report data only for the most commonly reported antigen (EBV viral capsid antigen, VCA), but state where other data were available. As there was substantial heterogeneity in study design, reporting and the risk factors examined, we present a narrative synthesis of our findings in place of a meta-analysis.

The quality of included studies was assessed using a checklist adapted from Downs and Black [15], as per the guidance issued by Deeks et al [16]. Sufficient adjustment for confounding was defined as adjusting for age, sex, ethnicity or country of birth, and some measure of socioeconomic status; this was a pragmatic decision based on common confounders and prior knowledge. For the purposes of the sample size calculation, age was treated as the main exposure of interest. As per Downs and Black, when assessing the power of studies, we calculated the minimum sample size per strata that would be required to detect a change in EBV seroprevalence from 95% to 85%, with power ranging from 70%-99%. Conservatively, we assumed only two strata and a ratio of 1:1 between exposure strata. Different thresholds were used for case-control studies and for cohort or cross-sectional studies. These criteria were scored from 0 (<70% power) to 5 (>99% power).

This review was registered on PROSPERO (CRD42017059811).

## RESULTS

### Search results and included studies

**Figure 1:**
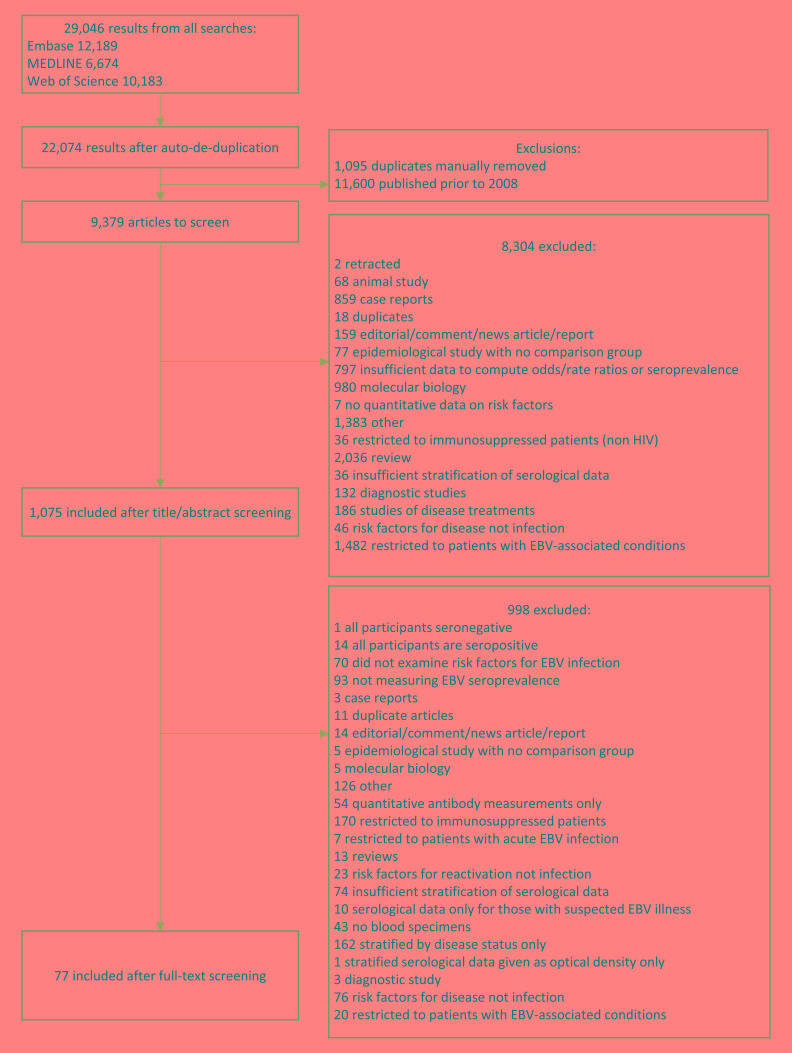
PRISMA flowchart of studies included in the systematic review.

**Figure 2:**
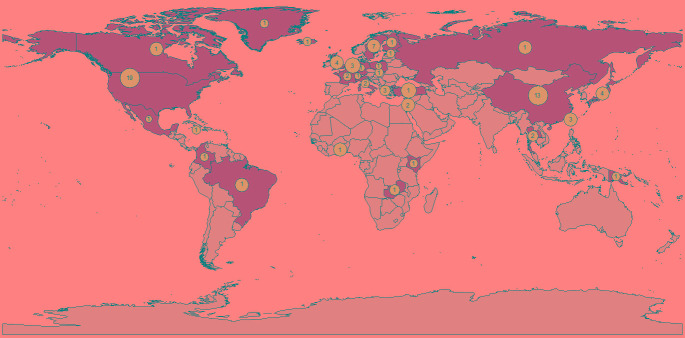
The global distribution of study populations included in the literature review. The size of the blue dots is proportional to the total number of individuals included in studies from each country. The number inside the dot refers to the number of studies with participants in each country.

Our literature search yielded 22 074 results after de-duplication. After exclusion of papers published before 2008 (n = 11 600), and title, abstract and full-text screening (**Figure 1**), 77 studies were included (Table S1 in the **Online Supplementary Document**). Three otherwise eligible studies were excluded; one as all participants were seropositive for EBV, one as only one patient was seronegative and one study which was a meta-analysis of the association between latitude and EBV seroprevalence which defined latitude based on the study location rather than participant-level data. Studies ranged in size from 8-61 273 participants, EBV seroprevalence ranged from 6%-91%. The global distribution of studies is shown in **Figure 2**. There were 12 case-control studies (3 nested within cohorts), 13 cohort/longitudinal and 16 which reported only cross-sectional results but were part of cohort. Thirty-three were cross-sectional, and the design of two other retrospective studies was unclear.

Study populations included population-level studies of healthy individuals (n = 42), pregnant women (n = 3) and healthy family members of people with NPC (n = 3), and studies of specific patient populations such as transplant recipients (n = 7), those infected with the human immunodeficiency virus (HIV, n = 5), and others (including haemophilia and heart disease, n = 17).

### Quality assessment of included studies

The results of our quality assessment are presented in Table S2 in the **Online Supplementary Document**. Thirty-eight studies examined EBV serostatus as their primary outcome and 39 as a secondary outcome. Only ten adequately adjusted for confounding, however there were no discernible differences between the results of these studies and the other studies included. Many studies lacked power; the impact of this is discussed throughout. Misclassification of serostatus may also have occurred as many studies used in-house, unvalidated assays for detecting EBV antibodies (Table S1 in the **Online Supplementary Document**); these studies tended to be smaller and were less likely to satisfy other quality criteria.

### Age and EBV seroprevalence

**Figure 3:**
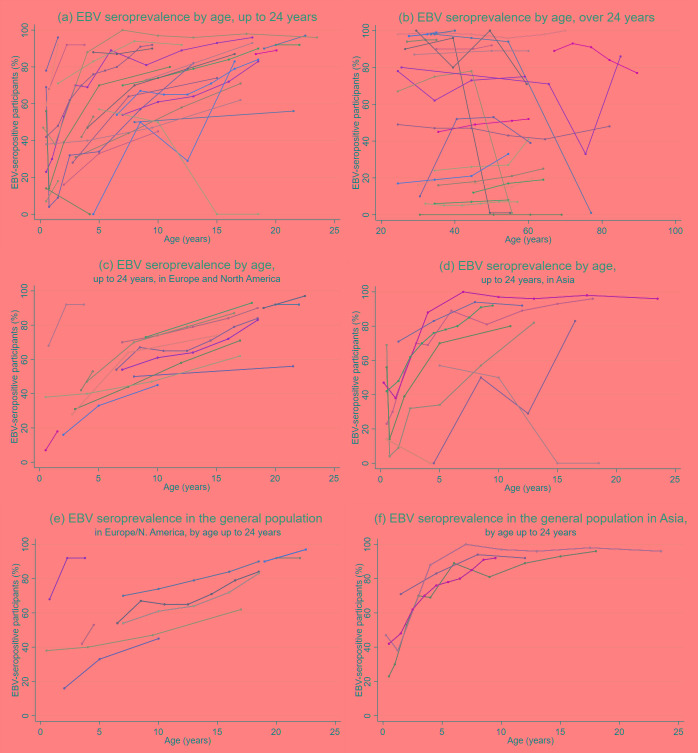
EBV seroprevalence by age in studies identified from the literature. **Panel A.** In participants up to 24 years of age. **Panel B.** In participants over 24 years of age. **Panel C.** In participants up to 24 years of age in Europe and North America. **Panel D.** In participants up to 24 years of age in Asia. **Panel E.** In participants up to 24 years of age in studies representative of their underlying populations in Europe and North America, (f) in participants up to 24 years of age in studies representative of their underlying populations in Europe and North America. Seroprevalence data categorised by age group was extracted from the literature and data points were plotted at the mid-point of each age group. Studies with only one data point above or below 24 years were excluded from the relevant graph. Studies were considered generalisable to the underlying population if it was a random sample of healthy individuals and the study population was representative of the local population.

Fifty-eight studies examined age as a risk factor for EBV serostatus; the majority (n = 32) found EBV seroprevalence increased with age (**Table 1**) [8,17-43,45-48]. There was a clear trend towards increasing EBV seroprevalence with age up to the age of 24 years (**Figure 3**), particularly when studies with <100 participants (n = 5) were excluded. Studies in older individuals (≥25 years) showed little change in seroprevalence.

**Table 1:** Summary of sociodemographic and lifestyle factors associated with EBV serostatus in the literature*

Risk factor for EBV serostatus	Summary of results
Age	Seronegativity decreased with age: 28 studies [8,17-44]
	Seronegativity decreased with age after 6 months: 3 studies [45-47]
	Seronegativity decreased with age after 18 months: 1 study [48]
	No association (children included): 4 studies [49-52]
	No association (all/vast majority adults): 12 studies [10,53-63]
Sex/gender	Women were more likely to be seronegative: 8 studies [22,49,57,59,62,64-66]
	Men were more likely to be seronegative: 6 studies [18,30,32,34,50,51]
	No association in children, but adult women were less likely to be seronegative: 1 study [26]
	Differences by sex interacted with marital status: 1 study [67]
	No association: 20 studies [8,17,19,20,23,29,42-44,54-56,60,61,63,68-72]
Ethnicity	Seroprevalence was lower for white participants than those of other ethnicities: 3 studies [20,43,71]
	EBV seronegativity was higher in people of Han ethnicity than other Chinese ethnicities: 1 study [57]
	No association: 7 studies [31,34,53,69,73-75]
Year of participation in study	No association: 2 studies [28,76]
Country of study	No difference between Japan and Jamaica: 1 study [77]
	Higher EBV seroprevalence in Mexico than Papua New Guinea, Columbia, Italy, Netherlands and Israel: 1 study [78]
Place of birth	EBV seronegativity was higher in central/Eastern China than Western China: 1 study [57]
	EBV seronegativity was higher among people of European/North American origin than other world regions: 2 studies [10,79]
	No association: 5 studies [19,20,36,37,55]
Socioeconomic status (SES)	EBV seronegativity was associated with higher SES: 1 study [10]
	No association between EBV and SES: 1 study [73]
	No association with occupational/social class: 3 studies [57,60,69]
	Higher household income was associated with EBV seronegativity: 2 studies [43,71]
	No association with household income: 1 study [20]
	EBV seronegativity was associated with having medical insurance for non-white participants: 1 study [43]
	No association with having private medical insurance: 1 study [75]
Level of education	Seronegativity increased with higher levels of education: 5 studies [10,19,20,43,57]
	No association with level of education of study participant: 2 studies [34,75]
	EBV seronegativity was higher among those whose parents had been in education for longer: 2 studies [10,71]
	No association with parental education: 1 study [8]
Anthroposophic lifestyle	No association: 1 study [24]
Urban/rural setting	EBV seronegativity lower in urban areas than rural areas: 1 study [10] No association: 1 study [21]
Household size/structure	***Number of siblings***
	EBV seroprevalence increased with number of siblings: 3 studies [10,66,71]
	No association between number of siblings and EBV seropositivity: 2 studies [8,55]
	***Birth order***
	No association with birth order: 2 studies [25,55]
	***Number of people in household***
	Adults with more children in the house were more likely to be EBV seropositive: 1 study [69]
	No association between number of adults in the house and EBV serostatus: 1 study [69]
	EBV seroprevalence increased with household size: 1 study [79]
	No association with household size: 1 study [53]
Crowding of home	No association: 4 studies [20,25,43,69]
Housing type (flat/house)	No association: 1 study [66]
Marital status	EBV seronegativity higher in unmarried women than married women, but lower in unmarried men than married men: 1 study [67]
	No association: 3 studies [19,53,57]
Sexual behaviour	EBV seroconversion was associated with deep kissing: 1 study [55]
Smoking status	***Smoking***
	Smoking associated with EBV seropositivity: 3 studies [10,39,79]
	Increased association with greater exposure: 1 study [39]
	No association: 6 studies [19,56,60,61,64,75]
	***Passive smoking***
	Mother smoking associated with lower EBV seronegativity: 1 study [66]
	No association: 1 study [25]
Weight/body mass index (BMI)	Increased BMI was associated with lower rates of seroprevalence: 4 studies [64,79-81]
	No association: 2 studies [71,75]
Diet	***General dietary factors***
	No association with diet: 1 study [55]
	No association with eating sufficient food: 1 study [82]
	No association with eating balanced meals: 1 study [82]
	No association with a reliance on low-cost food: 1 study [82]
	***Specific foods***
	No association with salted fish consumption: 3 studies [39,56,61]
	No association with frequency of fruit and vegetable consumption: 2 studies [56,82]
	No association with frequency of eating leafy salad: 1 study [82]
	No association with frequency of eating wholegrain bread: 1 study [82]
	No association with frequency of eating beans: 1 study [82]
	No association with frequency of eating red meat: 1 study [82]
	No association with betel nut consumption: 1 study [56]
	No association with slow-cooked soup consumption: 1 study [39]
	No association with preserved vegetable consumption: 1 study [39]
	***Specific drinks***
	No association with frequency of drinking milk: 1 study [82]
	No association with frequency of drinking juice: 1 study [82]
	No association with tea consumption: 1 study [39]
	No association with herbal tea consumption: 2 study [39]
Alcohol consumption	No association: 3 studies [19,39,56]
Formaldehyde/solvent exposure	No association: 1 study [56]
Exercise	No association: 1 study [55]
Height	No association: 2 studies [60,64]
Birth factors (baby)	***Vaginal vs caesarean delivery***
	No association: 3 studies [25,66,71]
	***Premature birth***
	No association: 3 studies [25,66,71]
	***Birth weight***
	No association: 3 studies [8,66,71]
Maternal characteristics	***Maternal parity***
	No association: 1 study [53]
	***Maternal age***
	No association: 2 studies [8,71]
	***Maternal smoking/alcohol use during pregnancy***
	No association: 1 study [71]
	***Maternal BMI prior to pregnancy***
	No association: 1 study [71]
	***Maternal fever during third trimester***
	No association: 1 study [71]
Stress	No association with stress: 1 study [55]
	No association with parental stress: 1 study [25]
Attended daycare	***Attendance***
	Attending daycare was associated with higher EBV seropositivity: 1 study [66]
	No association: 2 studies [20,71]
	***Age of starting daycare***
	Daycare attendance at a younger age was associated with greater EBV seropositivity: 1 study [25]
	No association: 1 study [66]
Hygiene practices	No association with frequency of house cleaning: 1 study [25] No association with frequency of handwashing: 1 study [25] No association with having pets in the house: 2 studies [25,66]
Swimming	No association with attending a swimming pool: 1 study [25]
Duration of watching television	No association with daily duration of watching television: 1 study [25]

BMI – body mass index, SES – socio-economic status, EBV – Epstein-Barr virus

*Adjusted results are presented where they were available, otherwise unadjusted associations are reported. There was some overlap in the data used by five studies based on data from the US National Health and Nutrition Examination Surveys [43,76,79,82,83]. To avoid over-representing the findings from this population, for each risk factor we have only included the findings of the largest study in this table.

When stratifying by age and study location and restricting the analysis to studies with populations representative of the general underlying population (n = 13 for participants <25 years), the data showed EBV infection occurred earlier in people in Asia than people in Europe and North America (**Figure 3**). In Asia EBV seroprevalence rapidly increased with age, exceeding 80% by age 5, and 90% by 7-8 years [19,21,36,47]. In contrast, studies in Europe and North America showed a more gradual increase in seroprevalence which did not exceed 90% until age 22 [23,25,28,33,38,43,49,53,79,83]. Studies which used validated, commercially available tests for EBV antigens were more likely to show increases in seroprevalence with age than studies which used in-house tests or did not reported which test they used; these studies also tended to be larger and representative of the underlying population (Table S1 in the **Online Supplementary Document**). Only thirteen studies fulfilled our power criteria across all age categories (Table S2 in the **Online Supplementary Document**); the remainder of the literature was consistent with the findings of these studies.

Most (12/16) studies that reported no association with age were of adult populations [10,53-63], suggesting that age only influences EBV seroprevalence during childhood.

### Other sociodemographic factors and EBV seroprevalence

The majority of studies (19/36) reported no association between sex and EBV serostatus (**Table 1**) [8,17,19,20,23,29,42,43,54-56,60,61,63,68-72]. Studies which reported associations [18,22,26,30,32,34,49-51,57,59,62,64-67] were evenly split in their findings, with no discernible differences according to study type, population or location, although they were generally small and lacking in power; 9/15 had ≤75 participants.

Relatively few studies (n = 11) compared EBV seroprevalence between participants of different ethnicities or countries of birth. The majority found no association [31,34,53,69,73-75], but three reported seroprevalence was lower among white participants than other ethnicities [20,43,71].

Multiple studies investigated associations between socioeconomic status and EBV seropositivity (**Table 1**). Five reported no association [20,57,60,69,73], whereas eight showed that EBV seropositivity was lower in people with higher levels of education [10,19,20,43,57], higher socioeconomic status [10], social class or household income [43,71]. All the studies reporting an association between lower EBV prevalence and higher socioeconomic status examined young people (≤21 years), whereas those that found no association examined all ages.

There was mixed evidence regarding an association with household size or daycare attendance (**Table 1**). Crowding of the home was not associated with EBV [20,25,43,69]. These findings did not differ by study size, design, location or population. Four studies found that smoking or passive smoking was associated with higher EBV seroprevalence [10,39,66,79]; one study reported a dose-response effect [39]. However, a further six studies reported no association [19,56,60,61,64,75]. Again, there were no discernible design differences between studies (Table S1 in the **Online Supplementary Document**).

Four of six studies reported that seroprevalence was higher among those who were overweight/obese than people of a healthy weight for older children and adults [64,79-81], whilst two studies of very young children and elderly women with physical difficulties reported no association [71,75]. Dietary factors were generally not associated with EBV seroprevalence (**Table 1**), nor were frequency of handwashing, house cleaning, or having pets in the house (**Table 1**).

### Viral and immune factors associated with EBV seroprevalence

Thirteen studies reported data on the correlation between EBV and CMV seroprevalence (**Table 2**); ten reported a positive association [31,35,48,60,66,83-87]; however studies which reported a negative association or no association tended to lack power [62,88].

**Table 2:** Summary of viral and clinical factors associated with EBV serostatus in the literature

Risk factor for EBV serostatus	Summary of results
CMV infection	Positive correlation between EBV and CMV serostatus: 10 studies [31,35,48,60,66,83-87]
	Negative association: 1 study [62]
	No association: 2 studies [88,89]
KSHV infection	Positive correlation between EBV and KSHV: 1 study [41]
HTLV infection	Positive correlation between EBV and HTLV serostatus: 1 study [77]
HSV-1 infection	Positive correlation between EBV and HSV-1 serostatus: 1 study [83]
Toxoplasmosis infection	No association: 1 study [89]
Rubella infection	No association: 1 study [89]
Syphilis	No association: 1 study [89]
Anti-IFN-gamma autoantibodies	No association: 1 study [89]
HIV infection	***HIV infection***
	Positive correlation between EBV and HIV status: 2 studies [8,17]
	No association: 1 study [90]
	***CD4 count***
	No association: 2 studies [54,57]
	Mother’s low CD4 percentage was associated with EBV seropositivity: 1 study [8]
	***Viral load***
	Mother’s higher viral load was associated with being EBV seropositive: 1 study [8]
Sensitised to IgE (allergy testing)	EBV-seronegative individuals had higher odds of ≥1 positive specific IgE test: 1 study [52]
	No association: 1 study [24]
Maternal family history of atopy	No association: 1 study [71]
Positive skin prick tests	EBV-seronegative individuals had higher odds of ≥1 skin prick test: 1 study [52]
Breastfed	***Breastfeeding***
	No association: 1 study [20]
	***Duration of being breastfed***
	No association with duration of being breastfed: 4 studies [8,25,66,71]
Respiratory or gastrointestinal tract infections in first year of life	No association: 1 study [71]
History of tonsillectomy	More common among EBV seronegative individuals: 1 study [73]

CMV – cytomegalovirus, EBV – Epstein-Barr virus, HIV – human immunodeficiency virus, HSV – herpes simplex virus, HTLV – human T-cell lymphotrophic virus, IFN – interferon, IgE – immunoglobulin E, KSHV – Kaposi’s sarcoma-associated herpesvirus

EBV seropositivity was also associated with infection with Kaposi’s sarcoma-associated herpesvirus (KSHV) [41], human T-cell lymphotropic virus (HTLV) [77], herpes simplex virus-1 (HSV-1) [83] and HIV [8,17]. CD4 count was not associated with EBV status in study participants with HIV (**Table 2**) [54,57], however, EBV seroprevalence was higher among infants whose mothers had HIV and were immunosuppressed [8].

There was some evidence of an association between EBV seroprevalence and positive allergy tests (**Table 2**) [52], although other studies found no association with positive allergy tests or atopy [24,71]. Prior tonsillectomy was less common among those who were EBV seropositive [73]. There was no association with having been breastfed [20], duration of breastfeeding [8,25,66,71], or having other gastrointestinal or respiratory tract infections during the first year of life [71].

### Genetic factors associated with EBV seronegativity

Three studies examined associations between immune system genes and EBV serostatus (**Table 3**). We refer here to associations with seronegativity, rather than seropositivity, as these are the genetic differences relevant to vaccination. Polymorphisms in the mannose-binding lectin gene [24], but not the defensin beta 1 gene [64], were associated with EBV seronegativity in children. Polymorphisms regulating the function of HLA-Bw4 and HLA-C were associated with EBV seronegativity in a cohort of healthy people aged >60 years [73]. Two other studies reported associations between gene polymorphisms and the EBV-specific antibody response in people that acquired the virus. None of these studies made adequate adjustments for confounding and their findings have not been replicated.

**Table 3:** Summary of human genetic factors associated with EBV serostatus in the literature

Risk factor for EBV serostatus	Summary of results
Immunological markers	HLA-C variant with the presence of TT at position -35 was more common in EBV seropositive individuals: 1 study [73]
	Frequency of HLA-Bw4 epitopes was lower in EBV seronegative individuals: 1 study [73]
	MBL-insufficient genotype (vs MBL-sufficient genotype) was associated with higher rates of EBV seronegativity: 1 study [23]
	No association between haplotype of the β-defensin-1 gene (*DEFB1*) and EBV seroprevalence: 1 study [91]

EBV – Epstein-Barr virus, C – cytosine, HLA – human leukocyte antigen, IL – interleukin. MBL – mannose-binding lectin, T – thymine

## DISCUSSION

Our systematic review of the literature is, to our knowledge, the first to summarise global risk factors for EBV infection whilst simultaneously assessing the quality of studies using a standardised tool. This formal assessment of the epidemiological evidence is critical to provide a comprehensive summary of the current evidence base to inform the direction of future research and policy, and to identify gaps that exist in the literature either because of a total absence of research, or an abundance of poor-quality research. Increasing age and co-infection with CMV were strongly associated with higher EBV seroprevalence. Critically, infection with EBV occurred at a younger age in study populations in Asia than in Europe or North America. Additionally, socioeconomic status, household size, smoking, body mass index (BMI), co-infections and genetic variants of immunological genes were associated with EBV serostatus, with varying degrees of certainty.

The age at which EBV infection occurs is an essential consideration when designing infection-preventing vaccination strategies. Variability in the age at infection between countries and settings will be a critical factor in deciding if, when, and how to roll out infection-preventing EBV vaccines in order to ensure cost-effectiveness, and whether or not a vaccine is cost-effective may differ between settings. Our literature review demonstrated that in Asian populations, where seroprevalence reached 80% by age 5, vaccinating infants would be essential to prevent infection. In contrast, in Europe and North America seroprevalence increases more gradually throughout childhood and adolescence, and the burden of IM is higher [3]. A vaccine like the original gp350-based based vaccine [92], which was unable to prevent infection but reduced IM incidence, could be deployed in these populations. Debilitating in its own right, IM is also associated with increased risk of developing EBV-positive Hodgkin’s lymphoma (HL) and multiple sclerosis (MS) [93,94], accordingly, a vaccine capable of reducing IM should decrease incidence of both conditions over time. A vaccine capable of preventing EBV infection from occurring could potentially have a much greater impact; reducing not only the incidence of HL but also of other malignancies where EBV infection is necessary for pathogenesis, but IM has not been reported to increase the risk. In addition to cancers, a history of IM is also associated with an elevated risk of multiple sclerosis; an IM-preventing vaccine could therefore potentially reduce the risk of this common autoimmune disease. However, a much greater effect might be achieved by a fully protective EBV vaccine, since the relative risk of MS development in EBV seronegative individuals is substantially lower than that of those carrying the virus [95]. Multiple lines of evidence support a causal role for EBV in MS [95]. If EBV infection is truly a pre-requisite for MS development then there is, in principle, no reason why a vaccine that prevents EBV infection would not prevent MS.

Importantly, incomplete vaccine coverage or imperfect efficacy could also increase the average age of EBV acquisition by reducing the pool of susceptible individuals, but not enough to prevent transmission; potentially increasing the likelihood of EBV-infection associated sequelae such as IM. Vaccination against Varicella Zoster virus (the only herpesvirus currently preventable by vaccination) is not recommended unless high vaccine coverage can be achieved, because increasing the average age of infection results in greater severity of disease [96]. The impact of age at EBV infection on the incidence of EBV-associated cancers is relatively unknown. For an EBV vaccination program to prevent EBV-associated cancers, vaccination policies will also be dependent on the rates of EBV-associated cancers in different settings, and the age at which these cancers typically occur.

There is a considerable lack of data on EBV seroprevalence by age in Africa and South America, and many studies from Asia were not generalisable to the wider population, thus limiting their usefulness for vaccination planning. High-quality, setting-specific data on the age at which EBV infection is acquired are essential for understanding EBV epidemiology in different settings and informing effective local vaccination policies. Future work is also required to understand why some individuals remain EBV seronegative for life, and the implications of this, which has important implications before implementing any vaccination strategy.

Many sociodemographic risk factors have been associated with EBV infection, however the causality of these relationships is difficult to determine for two broad reasons. First, socioeconomic status (SES) is a complex and dynamic measure that reflects different levels of exposure to multiple environmental and lifestyle factors. Second, lower SES is linked to diverse multiple biological factors that include chronic stress, increased pro-inflammatory immune factors [97], lower overall immunity and potentially shorter telomere length [98], although the causal nature of these relationships are not understood. Many of these factors could conceivably alter susceptibility to EBV infection and the risk of EBV infection occurring at a younger age. Dedicated studies exploring a wide range of sociodemographic and lifestyle factors within whole families are therefore needed to understand the causal factors leading to EBV infection in more detail, and these factors are likely to vary in different settings. Based on the current data it is, however, possible to make three general conclusions. First, the evidence showing SES was associated with EBV seroprevalence in studies of young people, but not in studies that included adults older than 21 years, suggests that SES is associated with the age at which EBV infection is acquired, but not with whether individuals remain uninfected for life. Thus, SES may have important effects on EBV-associated disease by mediating the age at which individuals are infected; it is known that EBV infection during adolescence frequently causes IM. Second, risk factors relating to SES which may directly influence the risk of EBV transmission (such as household size and crowding) were not found to be associated with EBV serostatus in our review, although it is possible they may be associated with the age of infection, which we were not able to explore. Finally, the mixed evidence as to whether there is an association between smoking and EBV suggests that this could be due to the association with socioeconomic status, rather than smoking being an independent risk factor. Similarly, higher income and/or social class, or higher BMI are likely confounders for other causal factors, rather than having an independent effect. However, what these causal factors may be remains unclear.

A meta-analysis of the association between EBV seroprevalence and latitude (of the studies, not individual participants) reported that EBV seroprevalence increased with distance from the equator, and suggested that as well as genetic, social and climactic factors, vitamin D may have a role in EBV-associated disease [99]. We did not identify any studies which looked at the association between vitamin D and EBV seroprevalence; this may merit further investigation.

There was a consistently positive association between infection with EBV and CMV, however we were unable to determine the temporality of this relationship, as no studies found in the literature have examined whether one infection preceded the other. The association may result from shared genetic, immunological and/or sociodemographic risk factors, or one infection could increase susceptibility to the other. Longitudinal studies with serial testing are necessary to explore this association in more detail. We note that a variety of antibodies were used to measure seroprevalence across the different studies, providing an additional degree of heterogeneity. Although IgG is the most utilised as an indicator of antibody-based immunity some studies also measured IgM (early-stage humoral immunity) and/or IgA (found in mucosal areas; IgA anti-VCA is more associated with nasopharyngeal carcinoma patients).

While multiple studies have studied links between genes involved in antiviral immunity and EBV-related disease [2], we identified only three studies examining links between such genes and EBV serostatus within the time period of our inclusion criteria. This small number of studies may be a consequence of the low frequency of EBV seronegative individuals in the adult population meaning large cohorts are required to identify sufficient EBV seronegative donors to produce robust conclusions. All three genes identified as being associated with EBV serostatus (mannose-binding lectin, HLA-Bw4 and HLA-C) are involved in the innate immune system; and older, smaller studies have reported similar associations [100-102]. Further studies are needed to determine the mechanisms by which these polymorphisms are associated with a lack of EBV infection, and whether other genes are similarly associated with EBV serostatus.

The comprehensive search strategy for the systematic review was designed to be highly sensitive, and we included papers in any language to ensure our results were comprehensive. Heterogeneity in study design in the existing literature, and wide variation in the risk factors reported, meant that a formal meta-analysis of our findings was not appropriate.

Confounding made it difficult to compare studies as there was substantial variation in the factors each paper adjusted for, and many studies only reported univariable results. However, this did not appear to cause systematic bias in the results. An important contribution of this study is that it will enable future studies to be better informed regarding the confounders they need to control for in their analyses in order to ensure unbiased estimates of effect sizes for other risk factors. The majority of studies were cross-sectional, meaning that the temporality of associations could not be assessed. As EBV infection is often acquired a young age, it may have preceded the reported risk factors, particularly factors such as BMI, smoking and diet. It is unclear whether high BMI is a risk factor for EBV infection, whether EBV infection predisposes individuals to obesity, or whether BMI is a confounder for other factors causally associated with EBV. The majority of the studies we found were conducted in Europe, North America and Southeast Asia, therefore our findings may not be generalisable outside these settings.

## CONCLUSIONS

In this systematic review we document the diverse factors that have been analysed globally for potential associations with EBV seroprevalence, provide a formal assessment of the quality of studies, and discuss the implications for future vaccine policy. EBV seroprevalence generally increases until around 24 years of age and remains constant thereafter; however infection is acquired earlier in Asia than in Europe and North America. Consequently, depending upon the efficacy and duration of protection of a vaccine, different vaccination strategies may be required in different settings. In Asia, vaccination of infants would be required to prevent EBV infection from occurring. In contrast, in Western countries a vaccine with limited duration of protection could prevent IM in older seronegative individuals, thereby reducing EBV-associated disease. There is a lack of high-quality data on the prevalence and age of EBV infection outside of Europe, North America and South-East Asia, which will be essential for informing effective vaccination policies in these settings.

## Additional material

Online Supplementary Document
