# Supplementary material for: Predictors of Epstein-Barr virus serostatus and implications for vaccine policy: A systematic review of the literature
Source: J Glob Health. 2020 Mar 30;10(1):010404. doi: 10.7189/jogh.10.010404 (PMC7125428; doi:10.7189/jogh.10.010404)
Supplement: Online Supplementary Document [file jogh-10-010404-s001.pdf]

Supplementary Materials

S1

Supplementary item 1: Systematic review search strategy and search results as conducted on 6th March 2017

Total de-duplicated results: 22,074

MEDLINE: total hits 6,647

1	herpesvirus 4, human.mp. or exp Herpesvirus 4, Human/	21977
2	epstein barr virus.mp.	30367
3	epstein-barr virus.mp.	30367
4	EBV.mp.	23035
5	EB virus.mp.	850
6	HHV-4.mp.	30
7	HHV4.mp.	17
8	HHV 4.mp.	30
9	exp Infectious Mononucleosis/ or infectious mononucleosis.mp.	8335
10	glandular fever.mp.	252
11	1 or 2 or 3 or 4 or 5 or 6 or 7 or 8 or 9 or 10	43267
12	seropositiv*.mp. [mp=title, abstract, original title, name of substance word, subject heading word, keyword heading word, protocol supplementary concept word, rare disease supplementary concept word, unique identifier, synonyms]	51517
13	seronegativ*.mp. [mp=title, abstract, original title, name of substance word, subject heading word, keyword heading word, protocol supplementary concept word, rare disease supplementary concept word, unique identifier, synonyms]	19437
14	exp Seroepidemiologic Studies/ or seroepidemiol*.mp.	22526
15	seroprevalence.mp.	16608
16	12 or 13 or 14 or 15	81420
17	epidemiol*.mp.	403097
18	exp Epidemiology/	23853
19	risk factor.mp. or exp Risk Factors/	763284
20	exp Cross-Sectional Studies/ or cross-sectional.mp.	342957
21	exp Cohort Studies/ or cohort.mp.	1784999
22	exp Case-Control Studies/ or case control.mp.	869702
23	case-control.mp.	265108
24	exp Clinical Trial/	781290
25	intervention study.mp.	7706

26	17 or 18 or 19 or 20 or 21 or 22 or 23 or 24 or 25	3505153
27	Humans/	16455048
28	16 or 26	3551245
29	11 and 27 and 28	6674

Embase: total hits 12,189

1	herpesvirus 4, human.mp. or exp Epstein Barr virus/	38145
2	epstein barr virus.mp.	46544
3	epstein-barr virus.mp.	46544
4	EBV.mp.	30679
5	eb virus.mp.	1090
6	hhv-4.mp.	50
7	hhv4.mp.	26
8	HHV 4.mp.	50
9	infectious mononucleosis.mp. or exp mononucleosis/	8941
10	glandular fever.mp.	205
11	1 or 2 or 3 or 4 or 5 or 6 or 7 or 8 or 9 or 10	57687
12	seropositiv*.mp.	40273
13	seronegativ*.mp.	20730
14	exp seroprevalence/ or exp seroepidemiology/ or seroepidemiol*.mp.	22636
15	12 or 13 or 14	68503
16	epidemiol*.mp. or exp epidemiology/	3468631
17	risk factor.mp. or exp risk factor/	968724
18	exp cross-sectional study/ or cross-sectional.mp.	362375
19	exp cohort analysis/ or cohort.mp.	654780
20	exp case control study/ or case control.mp.	182035
21	case-control.mp.	182035
22	clinical trial/	1041844
23	intervention study.mp. or exp intervention study/	47601
24	16 or 17 or 18 or 19 or 20 or 21 or 22 or 23	5223709
25	human/	18433133
26	15 or 24	5248213
27	11 and 25 and 26	12189

Web of Science (all databases): total hits 10,183

# 1	TS="human herpesvirus 4" OR TI="human herpesvirus 4"<br>*Timespan=All years*<br>*Search language=Auto*	10372
# 2	TS="epstein barr virus" OR TI="epstein barr virus"<br>*Timespan=All years*<br>*Search language=Auto*	58844
# 3	TS="epstein-barr virus" OR TI="epstein-barr virus"<br>*Timespan=All years*<br>*Search language=Auto*	58844
# 4	TS=EBV OR TI=EBV	37847

	Timespan=All years<br>*Search language=Auto*	
# 5	TS="eb virus" OR TI="eb virus"<br>*Timespan=All years*<br>*Search language=Auto*	1247
# 6	TS=hhv-4 OR TI=hhv-4<br>*Timespan=All years*<br>*Search language=Auto*	59
# 7	TS=hhv4 OR TI=hhv4<br>*Timespan=All years*<br>*Search language=Auto*	29
# 8	TS="hhv 4" OR TI="hhv 4"<br>*Timespan=All years*<br>*Search language=Auto*	59
# 9	TS="infectious mononucleosis" OR TI="infectious mononucleosis"<br>*Timespan=All years*<br>*Search language=Auto*	11563
# 10	TS="glandular fever" OR TI="glandular fever"<br>*Timespan=All years*<br>*Search language=Auto*	721
# 11	#10 OR #9 OR #8 OR #7 OR #6 OR #5 OR #4 OR #3 OR #2 OR #1<br>*Timespan=All years*<br>*Search language=Auto*	77011
# 12	TS=seropositiv* OR TI=seropositiv*<br>*Timespan=All years*<br>*Search language=Auto*	63974
# 13	TS=seronegativ* OR TI=seronegativ*<br>*Timespan=All years*<br>*Search language=Auto*	24943
# 14	TS=seroepidemiol* OR TI=seroepidemiol*<br>*Timespan=All years*<br>*Search language=Auto*	27066
# 15	TS=seroprevalence OR TI=seroprevalence<br>*Timespan=All years*<br>*Search language=Auto*	31725
# 16	#15 OR #14 OR #13 OR #12<br>*Timespan=All years*<br>*Search language=Auto*	110539
# 17	TS=epidemiol* OR TI=epidemiol*<br>*Timespan=All years*<br>*Search language=Auto*	2254515
# 18	TS="risk factor" OR TI="risk factor"<br>*Timespan=All years*<br>*Search language=Auto*	256204

# 19	TS="cross-sectional" OR TI="cross-sectional"<br>*Timespan=All years*<br>*Search language=Auto*	1092571
# 20	TS=cohort OR TI=cohort<br>*Timespan=All years*<br>*Search language=Auto*	658413
# 21	TS="case control" OR TI="case control"<br>*Timespan=All years*<br>*Search language=Auto*	288039
# 22	TS=case-control OR TI=case-control<br>*Timespan=All years*<br>*Search language=Auto*	291220
# 23	TS="clinical trial" OR TI="clinical trial"<br>*Timespan=All years*<br>*Search language=Auto*	329903
# 24	TS="intervention study" OR TI="intervention study"<br>*Timespan=All years*<br>*Search language=Auto*	10397
# 25	#24 OR #23 OR #22 OR #21 OR #20 OR #19 OR #18 OR #17<br>*Timespan=All years*<br>*Search language=Auto*	4229752
# 26	#25 OR #16<br>*Timespan=All years*<br>*Search language=Auto*	4277455
# 27	TS=human OR TI=human<br>*Timespan=All years*<br>*Search language=Auto*	24213522
# 28	#27 AND #26 AND #11<br>*Timespan=All years*<br>*Search language=Auto*	10183

S2

Supplementary table 1: Study characteristics of 77 studies of factors associated with EBV serostatus identified from the literature

Lead author	Reference	Year of publication	Study population	Number of participants	Study design	Country	Year of study	Age range	Percentage female	Socioeconomic position of study population (if stated)	Diagnostic test used	Type(s) of antibody detected	Definition of positive result	Overall EBV prevalence (%)
Abdollahi, A.	[52]	2014	People with and without HIV infection	228	Case-control*	Iran	2011-2012	Adults	30%	Middle-income country	ELISA (Mono Bind Inc.)	VCA IgG	>22 U/ml	61%
Adjei, AA.	[17]	2008	HIV-AIDS patients with chronic diarrhoea, and HIV-seronegative blood donors	3525	Cross-sectional	Ghana	2001-2002	17-65 years	23%	Developing country	ELISA (Advanced Biotechnologies)	VCA IgG	Cut-off values were calculated as per manufacturer's instructions	25%
Alcantara-Neves, NM.	[84]	2012	Children	1182	Cross-sectional nested within cohort	Brazil	2005	4-11 years	46%	Middle-income country	Immunoassay (Diamedix)	IgG	Manufacturer's instructions	89%
Balfour, HHJ.	[53]	2013	Freshmen in halls of residence at the University of Minnesota	546	Longitudinal	USA	2006-2007	18.0-22.1 years	60%	Wealthy country	EIA (Diamedix)	VCA and EBNA-1 IgG	≥1.10	63%
Balfour, HHJ.	[43]	2013	US non-institutionalised civilians aged 6-19 years	7516	Continuous cross-sectional survey	USA	2003-2010	6-19 years	49%	Wealthy country	Semi-quantitative enzyme immunoassay kits (Diamedix). Random sample compared to indirect immunofluorescence assay (Focus Laboratories) to assess concordance.	VCA IgG	Positive: EIA index ≥1.10	80%

Lead author	Reference	Year of publication	Study population	Number of participants	Study design	Country	Year of study	Age range	Percentage female	Socioeconomic position of study population (if stated)	Diagnostic test used	Type(s) of antibody detected	Definition of positive result	Overall EBV prevalence (%)
Bertrand, KA.	[67]	2010	Nurses and physicians	1002	Case-control nested within cohort	USA	1982-2003	Not stated	42%	Wealthy country	Indirect immunofluorescence and ELISA	VCA, EA, EBNA-1 and EBNA-2	Titer ≥1:20	95%
Bian, X.	[18]	2016	Healthy controls matched to cases of newly-diagnosed Type 1 diabetes	42	Case-control*	USA	Not stated	4-31 years	50%	Wealthy nation	RAPID ELISA (in-house)	IgG, IgA and IgM to VCA, EA and EBNA-1	Positive response to any EBV antigen	52%
Bigley, AB.	[78]	2012	Healthy adult men	20	Cross-sectional	USA	Not stated	22-35 years	0%	Wealthy country	ELISA (Genway Biotech)	IgG	Manufacturer's instructions	
Birmann, BM.	[83]	2009	Japanese and Jamaican adults	204	Case-control nested within cohort	Japan & Jamaica	1987-1993	≥28 years	82%	High and upper middle income countries	Immunofluorescence assay	VCA, EBNA-2 and EA	Titer ≥1:20	77%
Buckova, A.	[97]	2010	Healthy children and adolescents matched to patients with common variable immunodeficiency in Eastern Slovakia	394	case-control*	Slovakia	Not stated	2-18 years	43%	Poorer area of high-income country	ELISA (DiaSorin)	IgG & IgM to VCA, IgG to EBNA-1	Seronegative if antibody titre was <20AU/ml for all three antibodies	71%
Burbelo, PD.	[102]	2013	Immunodeficient patients with pathogenic anti-IFN-γ autoantibodies, Sjogrens syndrome or HIV infection, and healthy	137	Case-control nested within cohort*	Taiwan, Thailand, and USA	Not stated	Not stated	55%	Mixed	Luciferase Immunoprecipitation system	IgG	Not stated	98%

Lead author	Reference	Year of publication	Study population	Number of participants	Study design	Country	Year of study	Age range	Percentage female	Socioeconomic position of study population (if stated)	Diagnostic test used	Type(s) of antibody detected	Definition of positive result	Overall EBV prevalence (%)
			blood donors											
Chang, CM.	[54]	2012	Healthy people with >2 relatives with nasopharyngeal carcinoma	2393	Cross-sectional	Taiwan	Not stated	>18 years	53%	High-income country	Immunofluorescence assay, ELISA, enzyme neutralisation assay	VCA, EBNA-1, anti-Dnase	Anti-EBNA-1 IgG at OD405≥0.1	49%
Chen, CY.	[19]	2015	General population	1411	Cross-sectional	Taiwan	2007	All ages	59%	High-income country	ELISA (EUROIMMUN)	VCA IgG	≥22 RU/ml	89%
Condon, LM.	[20]	2014	Subjects with venous blood collected for medical indications at HealthPartners outpatient clinics in the Minneapolis-St Paul metropolitan area	705	Cross-sectional	USA	2011-2012	1.5-19.9 years	56%	Wealthy nation. Gives household income and education level.	Semi-quantitative enzyme immunoassay kits (Diamedix)	VCA IgG	Positive: EIA index ≥1.10	43%
Delaney, AS.	[48]	2015	US population	2857	Cross-sectional	USA	2003-2004	6-19 years	49%	Wealthy nation	Semi-quantitative enzyme immunoassay (Diamedix)	VCA IgG	EIA index ≥1.1	70%
Dowd, JB.	[50]	2013	US population	8417	Cross-sectional	USA	2003-10	6-19 years	48%	High income	Diamedix	VCA IgG	EIA index ≥1.1	67%
Du, HJ.	[21]	2008	Chinese children	589	Cross-sectional	China	Not reported	0-14 years	Not stated	Middle-income country	ELISA	VCA IgG	Manufacturer's instructions	84%
Du, JL.	[22]	2016	Chinese population without NPC	18286	Prospective longitudinal	China	1987 and 1992	30-59 years	62%	Middle-income country	Immunoenzymatic assay	VCA and EA IgG	antibody titer >1:5	7%
Durovic, B.	[73]	2013	Healthy blood donors	56	Cross-sectional	Switzerland	Not reported	>60 years	14%	Wealthy country	Multiplex microparticle technology (Luminex 200)	Not reported	Not reported	70%

Lead author	Reference	Year of publication	Study population	Number of participants	Study design	Country	Year of study	Age range	Percentage female	Socioeconomic position of study population (if stated)	Diagnostic test used	Type(s) of antibody detected	Definition of positive result	Overall EBV prevalence (%)
Ford, JL.	[98]	2013	American youths	6337	Cross-sectional	USA	2003-2010	6-17 years	49%	Wealthy country	EIA (Diamedix)	VCA IgG	EIA index ≥1.1	62%
Friborg, JT.	[23]	2010	Children in Sisimiut, Greenland	247	Prospective cohort	Greenland (Denmark)	1997-1998	0-4.5 years	54%	High income country	ELISA (Novitec)	VCA IgG and IgM	VCA-IgG >200 U/ml or VCA-IgM >500 U/ml	83%
Fuse, S.	[44]	2010	Patients with diagnosed or suspected Kawasaki disease	807	Case-control*	Japan	2004-2008	0-15 years	Not stated	Wealthy nation	ELISA	VCA IgG	Not stated	Not stated
Gerakari, S.	[68]	2011	Patients with HCV-related chronic liver disease	75	Retrospective	Greece	Not stated	19-70 years	48%	High income country	Not stated	VCA IgG and IgM	Not stated	68%
He, CS.	[62]	2013	Athletes at Loughborough university	236	Prospective cohort	UK	2011-2012	Not stated	30%	Wealthy country	ELISA (NovaTec Immunodiagnostica)	IgG	Manufacturer's instructions	84%
He, N.	[55]	2011	HIV/AIDS patients	1110	Cross-sectional	China	2008-2009	18-94 years	39%	Middle income country	ELISA (EUROIMMUN)	EBNA IgG	Manufacturer's instructions	97%
Hesla, HM.	[24]	2013	Children from anthroposophic and non-anthroposophic families	157	Prospective cohort	Sweden	2004-2007	0-2 years	49%	Wealthy country	Immunofluorescence assay	VCA IgG	Not stated	Not stated
Iju, XZ.	[45]	2011	Hospitalised children	14840	Cross-sectional	China	2009	0-15 years	19%	Middle income country	ELISA	VCA IgG and IgM	Manufacturer's instructions	49%
Jansen, MAE.	[65]	2016	Children in Rotterdam	4464	Prospective cohort	Netherlands	2002-2012	0-6 years	48%	Wealthy nation	Enzyme immunoassay (EUROIMMUN)	VCA IgG	Optical density >10% over manufacturers threshold	51%
Jeziorski, E.	[40]	2008	Age-matched controls of children with Langerhans	206	Case-control*	France	Not stated	0.5-20 years	Not stated	Wealthy nation	ELISA (DiaSorin)	VCA IgG & IgM	Above upper limit of "grey" zone	47%

Lead author	Reference	Year of publication	Study population	Number of participants	Study design	Country	Year of study	Age range	Percentage female	Socioeconomic position of study population (if stated)	Diagnostic test used	Type(s) of antibody detected	Definition of positive result	Overall EBV prevalence (%)
			cell histiocytosis											
Karachaliou, M.	[25]	2016	Live singleton births (mothers recruited)	81 to 690	Cohort	Greece	2007-08	0-4 years	Not stated	High income	In house fluorescence bead based multiplex serology	VCA, EBNA-1, ZEBRA, EA IgG	At least two Ags, mean seroreactivity of sero-ves plus 3 times SD	Not stated
Karadag, GM.	[26]	2014	Patients in Turkey	6822	Cross-sectional	Turkey	2009-2012	Children and adults	46%	Upper-middle income country	Enzyme-linked Fluorescent Assay (BioMerieux)	VCA IgG and IgM	Not stated	78%
Khandaker, GM.	[63]	2014	Children born to mothers in county Avon	530	Cross-sectional within cohort	England	1995-1996	4 years	58%	Wealthy nation	Indirect immunofluorescence	VCA IgG	Not reported	25%
Kucharska, M.	[27]	2016	HCV-infected hemophiliacs	71	Cross-sectional	Poland	Not stated	Not stated	6%	High-income country	ELISA (NovaTec)	VCA IgG	Not reported	96%
Levine, H.	[10]	2012	Israeli men recruited to mandatory military service	1249	Cross-sectional and prospective longitudinal	Israel	1994-2004	17.6-21.1 years	0%	high-income country	ELISA (NovaTec)	VCA IgG	Absorbance value >10% above cut-off	87%
Michos, A.	[74]	2011	Roma and non-Roma schoolchildren	216	Case-control*	Greece	2002	5-15 years	64%	High-income country	ELISA	Not stated	Not stated	48%
Minhas, V.	[41]	2010	Zambian infants who survived to the age of 2	677	Cohort	Zambia	1998-2004	1 year	49%	Developing country	ELISA (Diagnostic Automation)	VCA IgG	Not stated	59%
Mousavi, SSB.	[56]	2011	Renal allograft recipients	60	Cross-sectional	Iran	2009-2010	19-68 years	38%	Upper middle income country	ELISA	IgG	Not stated	70%
Naenifaerd, H.	[100]	2015	US children	1550	Cross-sectional	USA	2009-2010	6-15 years	49%	Wealthy nation	enzyme immunoassay	VCA IgG	EIA >1.10	56%
Pembrey, L.	[49]	2013	Pregnant women in Bradford	949	Cross-sectional within cohort	UK	2008-2010	>20 years	100%	Wealthy nation	Indirect chemiluminescence immunoassay	VCA IgG	Not stated	94%

Lead author	Reference	Year of publication	Study population	Number of participants	Study design	Country	Year of study	Age range	Percentage female	Socioeconomic position of study population (if stated)	Diagnostic test used	Type(s) of antibody detected	Definition of positive result	Overall EBV prevalence (%)
											(DiaSorin Liaison Analyser)			
Pordeus, V.	[101]	2008	Healthy volunteers in Colombia and Italy	240	Cross-sectional	Colombia and Italy	Not stated	18-63 years	82%	Middle & high-income countries	Bio-Rad BioPlex 2200 immunoassay	VCA, EBNA-1 and EA IgG	Three cutoff levels	83%
Pourahamad, M.	[72]	2014	Iranian men and women who were married or planning to marry	160	Case-control*	Iran	Not stated	Not stated	51%	Upper middle income country	ELISA (Diesse)	VCA IgG	Manufacturer's instructions	83%
Preiksaitis, JK.	[47]	2010	Solid-organ transplant recipients (pre-transplantation)	2935	Cross-sectional	Canada	1994-2007	0->39 years	Not stated	wealthy country	Enzyme immunoassay (Gull Laboratories or Captia)	VCA IgG or EBNA-1 IgG	Positive for both antibodies	95%
Puhakka, L.	[28]	2016	Pregnant wome	600	Cross-sectional within cohort	Finland	1992, 2002, 2012	Adults	100%	wealthy country	Enzygnost Anti-EBV/IgG	IgG	Manufacturer's instructions	97%
Qin, HD.	[29]	2011	Healthy family members of NPC patients, and healthy controls from two populations (one high-risk for NPC and one low-risk)	3395	Cross-sectional	China	1999-2005	All ages	49%	middle-income country	Guangdon Zhonshan company	VCA IgA	Not reported	27%
Rahimzadeh, N.	[64]	2013	Children with renal transplants	183	Retrospective	Iran	2003-2011	≤18 years	40%	Upper middle income country	Not stated	Not stated	Not reported	61%
Ritter, J.	[30]	2015	Allogeneic stem cell donors	23	Cross-sectional	Germany	Not reported	20-61 years	13%	wealthy country	CMIA assay	Not stated	Not reported	83%
Rodriguez, TA.	[51]	2010	Twins in a cohort for	28	Cohort*	USA	Unknown	6-72 years	75%	High income	Unknown	IgG	Unknown	68%

Lead author	Reference	Year of publication	Study population	Number of participants	Study design	Country	Year of study	Age range	Percentage female	Socioeconomic position of study population (if stated)	Diagnostic test used	Type(s) of antibody detected	Definition of positive result	Overall EBV prevalence (%)
			an alopecia study											
Rubicz, R.	[57]	2011	Extended Mexican American families participating in San Antonio Family Heart Study	1227	Cohort*	USA	1991-95 (recruitment)	15-94 years	61%	High income	Commercial ELISA	EBNA-1 and EA IgG	≥1.10	46%
Saghafi-Hendengren, S.	[79]	2013	Children whose parents have a history of allergy	128	Cohort	Sweden	2002-2005	5 years	Not stated	High income	Unknown	Not stated	Unknown	59%
Saghafi-Hendengren, S.	[69]	2009	Children whose parents have a history of allergy	51	Cohort	Sweden	1999-2002	2 years	49%	High income	In house	VCA IgG	1:20	49%
Saghafi-Hendengren, S.	[80]	2010	Children whose parents have a history of allergy	219	Cohort	Sweden	2002-2005	5 years	48%	High income	In house	VCA IgG	1:20	20%
Sampaio, MS.	[31]	2012	Kidney transplant recipients with known donor serostatus	61273	Cohort*	USA	1994-2010	Not stated	60%	High income	Unknown	Not stated	Unknown	87%
Sato, T.	[32]	2008	Renal transplant recipients	22	Cross-sectional	Japan	2002-2006	3.4-17.8 years	59%	Wealthy nation	Not stated	Not stated	Not stated	41%
Sato, T.	[70]	2008	Kidney transplant recipients	21	Cohort*	Japan	1997-2005 plus up to 8 yrs FU	3-19 years	48%	High income	Indirect immunofluorescence	VCA IgG and IgM, EA EgG, EBNA (not stated)	≥1.10	33%
Savva, GM.	[58]	2013	Older individuals	489	Cohort*	UK	Unknown	65-94 years	51%	High income	Demeditc	VCA IgG	>12U/ml	89%

Lead author	Reference	Year of publication	Study population	Number of participants	Study design	Country	Year of study	Age range	Percentage female	Socioeconomic position of study population (if stated)	Diagnostic test used	Type(s) of antibody detected	Definition of positive result	Overall EBV prevalence (%)
			without severe cognitive or physical impairments included in ESCR Healthy Aging Study											
Shapira, Y.	[99]	2012	Blood donors	557	Cross-sectional	Papua New Guinea, Colombia, Mexico, Italy, Netherlands, Israel	Unknown	Not stated	Not stated	Middle and high income	Bio-Rad BioPlex	VCA, EBNA-1, EA IgG	Unknown	Not stated
Shen, GP.	[59]	2011	Healthy individuals	755	Cross-sectional	China	2005-07	Not stated	26%	High income	Guangdong Zhongshan Company	VCA IgA	<1:10	83%
Simon, KC.	[33]	2012	Children whose parents have a history of allergy	154	Cohort	Sweden	2002-2010	10 years	Not stated	High income	Immunofluorescence	VCA IgG	Unknown	45%
Slyker, JA.	[8]	2013	Infants whose mothers have HIV	125	Prospective cohort	Kenya	2004-2008?	0-2 years	43%	Developing country	PCR and ELISA (Wampole)	VCA IgM and IgG, EBNA-1 IgG	Positive for EBV DNA or ≥1 EBV antibody (threshold not defined)	60%
Spielmann, G.	[76]	2014	Healthy Mexican-American children in an urban school	123	Cross-sectional	USA	Unknown	10-14 years	54%	High income	GenWay Biotech	IgG, unknown	Unknown	51%
Stowe, RP.	[34]	2010	Hispanic families living close to petrochemical plants	1457	Cohort*	USA	Unknown	Not stated	55%	High income	Microgen	VCA IgG	>1:1280	16%

Lead author	Reference	Year of publication	Study population	Number of participants	Study design	Country	Year of study	Age range	Percentage female	Socioeconomic position of study population (if stated)	Diagnostic test used	Type(s) of antibody detected	Definition of positive result	Overall EBV prevalence (%)
Stowe, RP.	[82]	2014	Pregnant Hispanic women	398	Cross-sectional	USA	Unknown	14-45 years	100%	High income	Bion	VCA IgG	Unknown	99%
Sundqvist, E.	[35]	2014	Population controls matched by age, gender and residential area to multiple sclerosis cases	786	Case-control*	Sweden	2005-09	16-70 years	74%	High income	Biotest	EBNA-1 IgG	Based on median among controls	50%
Suntornlohanakul, R	[46]	2015	Participants in study of universal HBV vaccination	583	Cross-sectional	Thailand	2014	0-57 years	50%	Middle income	EUROIMMUN	VCA IgG (and IgM if <2yrs)	<22 RU/ml	88%
Tesse, R.	[103]	2009	Control cases matched to children with acute lymphoblastic leukaemia	40	Case-control	Italy	2005-2007	1-14 years	43%	Wealthy country	ELISA (Meridian Diagnostic)	IgG	Not reported	53%
Thjodleifsson, B.	[77]	2008	Randomly selected individuals in age range 20-44 at start	985	Cohort*	Iceland, Sweden, Estonia	1999-2001	28-55 years	Not stated	High income	Unknown	IgG	Unknown	90%
Thomas de Montpreville, V.	[61]	2015	Individuals who developed post-transplant lymphoproliferative disease	16	Cohort*	France	2001-14	15-63 years	50%	High income	Unknown	VCA IgG	'Beyond normal levels'	75%
van den Heuval, D.	[81]	2016	Study of childhood,	1079	Cohort*	Netherlands	Unknown	5-7.9 years	Unknown	High income	EUROIMMUN	VCA IgG	Sample-threshold	47%

Lead author	Reference	Year of publication	Study population	Number of participants	Study design	Country	Year of study	Age range	Percentage female	Socioeconomic position of study population (if stated)	Diagnostic test used	Type(s) of antibody detected	Definition of positive result	Overall EBV prevalence (%)
			starting pre-birth										ratio above 0.8	
Wang, C.	[60]	2014	Healthy people	27	Cohort*	USA	2008-09	20-89 years	63%	High income	Calbiotech	IgG and IgM	Unknown	67%
Wang, GC.	[75]	2016	Women with difficulties in physical function dwelling in the community	633	Cohort*	USA	1992 onwards	70-79 years	100%	High income	GenWay Biotech	IgG	Ab index >1.1	73%
Wu, JF.	[71]	2009	Children who received liver transplants and developed post-transplant lymphoproliferative disorder	8	Cohort*	China	1997-2007	0-17 years	50%	High income	Unknown	EBNA	Unknown	13%
Xiong, G.	[36]	2014	Individuals who had undergone health and nutrition examinations	1778	Cross-sectional	China	2012-13	0-10 years	49%	High income	EUROIMMUN	VCA IgG/IgM, EA IgG, EBNA IgG	Unknown	78%
Xu, FH.	[39]	2012	Healthy men in Yangquan	1961	Cross-sectional	China	2010	16-87 years	0%	Middle income country	Immunoturbidimetry method (Hitachi)	VCA IgA	≥1.10	7%
			Healthy men in Guangdong	3228	Cross-sectional	China	2008	18-67 years	0%	Middle income country	Immunoturbidimetry method (Hitachi)	VCA IgA	≥1.10	18%
Yi, B.	[37]	2009	Healthy individuals attending physical examinations	395	Cross-sectional	China	Unknown	20-60 years	Not stated	High income	Sinoclone	VCA IgG and IgA, EBNA-1 IgG and IgA, Zta IgG and IgA	Adjusted relative absorbance ≥1	22%

Lead author	Reference	Year of publication	Study population	Number of participants	Study design	Country	Year of study	Age range	Percentage female	Socioeconomic position of study population (if stated)	Diagnostic test used	Type(s) of antibody detected	Definition of positive result	Overall EBV prevalence (%)
Yu, X.	[66]	2011	First-degree relatives of nasopharyngeal carcinoma patients and normal population	16686	Case-control*	China	2009.08-2010.07	30-59 years	57%	Middle income country	Not stated	VCA and EBNA-1 IgA	Not stated	6.8% in cases and 5.8% in controls
Yu, X.	[42]	2011	Healthy residents	16355	Cross-sectional	China	Not reported	30-59 years	57%	Middle income country	ELISA	VCA and EBNA-1 IgA	rOD≥1.000	40%
Zebrun, AB.	[38]	2013	Adult healthy blood donors, and school children	2322	Cross-sectional	Russia	2006-2012	0-62 years	49%	High income country.	ELISA	EA and EBNA	Not stated	29%

* Treated as cross-sectional data in our analysis

S3

Supplementary table 2: Quality checklist

Lead author	Ref.	Study of risk factors for EBV infection?	Is the study design clearly reported?	Is the hypothesis/ aim/ objective of the study clearly described?	Are the main outcomes to be measured clearly described in the Introduction or Methods section?	Are the characteristics of the patients included in the study clearly described?	Are the distributions of principal confounders in each group of subjects to be compared clearly described?	Are the main findings of the study clearly described?	Does the study provide estimates of the random variability in the data for the main outcomes?	Have the characteristics of patients lost to follow-up been described?	Have actual probability values been reported for the main outcomes except where the probability value is <0.001?	Was there potential for recall bias in the ascertainment of the exposure?	Was there potential for differential or non-differential misclassification of the exposure?	Was there potential for observer bias in ascertainment of the outcome?	Was there potential for differential or non-differential misclassification of the outcome?	In trials and cohort studies, do the analyses adjust for different lengths of follow-up of patients, or in case-control studies, is the time period between the intervention and outcome the same for cases and controls?	Were the statistical tests used to assess the main outcomes appropriate?	Were the main outcome measures used accurate (valid and reliable)?	Were the patients in different intervention groups (trials and cohort studies) or were the cases and controls (case-control studies) recruited from the same population?	Were study subjects in different intervention groups (trials and cohort studies) or were the cases and controls (case-control studies) recruited over the same period of time?	Was there adequate adjustment for confounding in the analyses of interest?	Were losses of patients to follow-up taken into account?	Are the study results appropriately interpreted e.g. in terms of the strength of the evidence, its application/ implications, causality?	Did the study have sufficient power to detect a clinically important effect where the probability value for a difference being due to chance is less than 5%?	Total
Abdollahi, A.	[52]	Yes	Yes	Yes	Yes	Yes	No	Yes	No	N/A	Yes	No	UTD	No	No	N/A	Yes	Yes	N/A	N/A	No	N/A	No	0	12
Adjei, AA.	[17]	Yes	Yes	Yes	Yes	Yes	Partially	Yes	No	N/A	No	No	UTD	No	No	N/A	Yes	Yes	N/A	N/A	No	N/A	Yes	2	16
Alcantara-Neves, NM.	[84]	No	Yes	Yes	No	Yes	Partially	Yes	Yes	N/A	No	No	No	No	No	N/A	Yes	Yes	N/A	N/A	No	N/A	N/A	4	16
Balfour, HHJ.	[53]	Yes	Yes	Yes	Yes	Yes	No	Yes	No	No	No	No	No	No	No	Yes	Yes	Yes	N/A	N/A	No	No	Yes	N/A	15
Balfour, HHJ.	[43]	Yes	Yes	Yes	Yes	Yes	Yes	Yes	Yes	N/A	No	No	No	No	No	N/A	Yes	Yes	N/A	N/A	Yes	N/A	Yes	5	23
Bertrand, KA.	[67]	No	Yes	Yes	No	Yes	Partially	Yes	No	N/A	No	No	No	UTD	No	N/A	No	UTD	N/A	N/A	No	N/A	N/A	N/A	9
Bian, X.	[18]	No	Yes	Yes	Yes	Yes	Partially	Yes	No	N/A	Yes	No	No	No	No	N/A	No	No	N/A	N/A	No	N/A	N/A	0	11
Bigley, AB.	[78]	No	Yes	Yes	Yes	Yes	Partially	Yes	No	N/A	No	No	No	No	No	N/A	Yes	Yes	N/A	N/A	No	N/A	N/A	N/A	12
Birmann, BM.	[83]	No	Yes	Yes	No	No	No	No	No	N/A	Yes	No	No	No	No	N/A	Yes	UTD	N/A	N/A	No	N/A	N/A	N/A	8
Buckova, A.	[97]	No	Yes	Yes	Yes	Yes	No	Yes	Yes	N/A	Yes	No	No	No	No	N/A	Yes	Yes	Yes	UTD	No	N/A	Yes	5	21
Burbelo, PD.	[102]	No	Yes	Yes	No	No	No	No	No	N/A	No	No	UTD	UTD	No	N/A	Yes	Yes	N/A	N/A	No	N/A	N/A	N/A	7

Lead author	Ref.	Study of risk factors for EBV infection?	Is the study design clearly reported?	Is the hypothesis/ aim/ objective of the study clearly described?	Are the main outcomes to be measured clearly described in the Introduction or Methods section?	Are the characteristics of the patients included in the study clearly described?	Are the distributions of principal confounders in each group of subjects to be compared clearly described?	Are the main findings of the study clearly described?	Does the study provide estimates of the random variability in the data for the main outcomes?	Have the characteristics of patients lost to follow-up been described?	Have actual probability values been reported for the main outcomes except where the probability value is <0.001?	Was there potential for recall bias in the ascertainment of the exposure?	Was there potential for differential or non-differential misclassification of the exposure?	Was there potential for observer bias in ascertainment of the outcome?	Was there potential for differential or non-differential misclassification of the outcome?	In trials and cohort studies, do the analyses adjust for different lengths of follow-up of patients, or in case-control studies, is the time period between the intervention and outcome the same for cases and controls?	Were the statistical tests used to assess the main outcomes appropriate?	Were the main outcome measures used accurate (valid and reliable)?	Were the patients in different intervention groups (trials and cohort studies) or were the cases and controls (case-control studies) recruited from the same population?	Were study subjects in different intervention groups (trials and cohort studies) or were the cases and controls (case-control studies) recruited over the same period of time?	Was there adequate adjustment for confounding in the analyses of interest?	Were losses of patients to follow-up taken into account?	Are the study results appropriately interpreted e.g. in terms of the strength of the evidence, its application/ implications, causality?	Did the study have sufficient power to detect a clinically important effect where the probability value for a difference being due to chance is less than 5%?	Total
Chang, CM.	[54]	Yes	Yes	Yes	No	Yes	Yes	Yes	Yes	N/A	Yes	No	No	UTD	No	N/A	Yes	UTD	N/A	N/A	Yes	N/A	Yes	5	22
Chen, CY.	[19]	Yes	Yes	Yes	Yes	Yes	Partially	Yes	Yes	N/A	Yes	No	No	No	No	N/A	Yes	Yes	N/A	N/A	No	N/A	Yes	0	17
Condon, LM.	[20]	Yes	Yes	Yes	Yes	Yes	Yes	Yes	Yes	N/A	Yes	No	No	No	No	N/A	Yes	Yes	N/A	N/A	No	N/A	Yes	1	18
Delaney, AS.	[48]	Yes	Yes	Yes	Yes	Yes	Yes	Yes	Yes	N/A	Yes	No	No	No	No	N/A	Yes	Yes	N/A	N/A	Yes	N/A	Yes	4	23
Dowd, JB.	[50]	Yes	Yes	Yes	Yes	Yes	Yes	Yes	Yes	N/A	No	No	No	No	No	N/A	Yes	Yes	N/A	N/A	Yes	N/A	Yes	N/A	18
Du, HJ.	[21]	Yes	Yes	Yes	Yes	Yes	Yes	Yes	Yes	N/A	No	No	No	No	No	N/A	No	Yes	N/A	N/A	No	N/A	Partially	0	15
Du, JL.	[22]	No	Yes	Yes	No	Yes	Partially	Yes	No	N/A	Yes	No	No	UTD	No	N/A	Yes	UTD	N/A	N/A	No	N/A	N/A	5	16
Durovic, B.	[73]	No	No	Yes	No	Yes	Partially	No	No	N/A	Yes	No	UTD	UTD	No	N/A	UTD	UTD	N/A	N/A	No	N/A	N/A	N/A	7
Ford, JL.	[98]	Yes	Yes	Yes	Yes	Yes	Yes	Yes	No	N/A	Yes	No	No	No	No	N/A	Yes	Yes	N/A	N/A	No	N/A	Yes	N/A	17
Friborg, JT.	[23]	Yes	Yes	No	Yes	Yes	Partially	Yes	No	No	Yes	No	No	No	No	UTD	Yes	Yes	Yes	UTD	No	No	Yes	0	16
Fuse, S.	[44]	No	Yes	No	No	No	No	Yes	No	N/A	No	No	UTD	UTD	No	N/A	UTD	UTD	N/A	N/A	No	N/A	N/A	0	5
Gerakari, S.	[68]	No	Yes	Yes	No	Yes	No	Yes	No	N/A	No	No	UTD	UTD	No	N/A	No	UTD	N/A	N/A	No	N/A	N/A	N/A	7
He, CS.	[62]	No	Yes	Yes	No	Yes	No	Yes	No	No	Yes	No	No	No	No	No	Yes	Yes	N/A	N/A	No	No	N/A	N/A	11
He, N.	[55]	Yes	Yes	Yes	Yes	Yes	Yes	Yes	No	N/A	Yes	No	No	No	No	N/A	Yes	Yes	N/A	N/A	UTD	N/A	Yes	0	17

Lead author	Ref.	Study of risk factors for EBV infection?	Is the study design clearly reported?	Is the hypothesis/ aim/ objective of the study clearly described?	Are the main outcomes to be measured clearly described in the Introduction or Methods section?	Are the characteristics of the patients included in the study clearly described?	Are the distributions of principal confounders in each group of subjects to be compared clearly described?	Are the main findings of the study clearly described?	Does the study provide estimates of the random variability in the data for the main outcomes?	Have the characteristics of patients lost to follow-up been described?	Have actual probability values been reported for the main outcomes except where the probability value is <0.001?	Was there potential for recall bias in the ascertainment of the exposure?	Was there potential for differential or non-differential misclassification of the exposure?	Was there potential for observer bias in ascertainment of the outcome?	Was there potential for differential or non-differential misclassification of the outcome?	In trials and cohort studies, do the analyses adjust for different lengths of follow-up of patients, or in case-control studies, is the time period between the intervention and outcome the same for cases and controls?	Were the statistical tests used to assess the main outcomes appropriate?	Were the main outcome measures used accurate (valid and reliable)?	Were the patients in different intervention groups (trials and cohort studies) or were the cases and controls (case-control studies) recruited from the same population?	Were study subjects in different intervention groups (trials and cohort studies) or were the cases and controls (case-control studies) recruited over the same period of time?	Was there adequate adjustment for confounding in the analyses of interest?	Were losses of patients to follow-up taken into account?	Are the study results appropriately interpreted e.g. in terms of the strength of the evidence, its application/ implications, causality?	Did the study have sufficient power to detect a clinically important effect where the probability value for a difference being due to chance is less than 5%?	Total
Hesla, HM.	[24]	No	Yes	Yes	No	Yes	No	Yes	No	No	Yes	No	No	UTD	No	Yes	Yes	UTD	Yes	Yes	No	No	N/A	1	14
Iju, XZ.	[45]	Yes	Yes	Yes	Yes	Yes	Yes	Yes	Yes	N/A	No	No	No	No	No	N/A	Yes	Yes	N/A	N/A	No	N/A	Partially	5	21
Jansen, MAE.	[65]	Yes	Yes	Yes	Yes	Yes	Yes	Yes	No	No	Yes	No	No	No	No	Yes	Yes	Yes	N/A	N/A	No	No	Yes	N/A	18
Jeziorski, E.	[40]	No	Yes	Yes	Yes	Yes	Partially	Yes	No	N/A	Yes	No	No	No	No	N/A	No	Yes	N/A	N/A	No	N/A	Yes	1	15
Karachaliou, M.	[25]	Yes	Yes	Yes	Yes	Yes	No	Yes	Yes	No	No	No	No	No	No	Yes	Yes	No	Yes	Yes	Yes	Yes	Yes	0	19
Karadag, GM.	[26]	Yes	No	Yes	No	Yes	Partially	Yes	No	N/A	No	No	UTD	No	No	N/A	No	Yes	N/A	N/A	No	N/A	No	5	14
Khandaker, GM.	[63]	No	Yes	Yes	No	Yes	Yes	Yes	No	N/A	Yes	No	No	UTD	No	N/A	Yes	UTD	N/A	N/A	No	N/A	N/A	N/A	12
Kucharska, M.	[27]	No	No	Yes	No	No	No	Yes	No	N/A	Yes	No	UTD	No	No	N/A	Yes	Yes	N/A	N/A	No	N/A	N/A	0	8
Levine, H.	[10]	Yes	Yes	Yes	Yes	Yes	Yes	Yes	Yes	N/A	Yes	No	No	No	No	N/A	Yes	Yes	N/A	N/A	Yes	N/A	Yes	4	23
Michos, A.	[74]	No	Yes	Yes	No	Yes	No	Yes	No	N/A	Yes	No	No	UTD	No	N/A	Yes	Yes	N/A	N/A	No	N/A	N/A	N/A	11
Minhas, V.	[41]	Yes	Yes	Yes	No	Yes	No	Yes	No	No	No	No	UTD	No	No	N/A	Yes	Yes	N/A	N/A	No	No	Yes	5	17
Mousavi, SSB.	[56]	No	Yes	Yes	No	Yes	No	Yes	No	N/A	No	No	No	No	No	N/A	No	Yes	N/A	N/A	No	N/A	N/A	0	9
Naenifaerd, H.	[100]	Yes	Yes	Yes	No	Yes	No	Yes	Yes	N/A	No	No	No	No	No	N/A	Yes	Yes	N/A	N/A	Yes	N/A	Yes	N/A	15
Pembrey, L.	[49]	Yes	Yes	Yes	No	Yes	Yes	Yes	No	N/A	Yes	No	No	No	No	N/A	Yes	Yes	N/A	N/A	Yes	N/A	Yes	0	17

Lead author	Ref.	Study of risk factors for EBV infection?	Is the study design clearly reported?	Is the hypothesis/ aim/ objective of the study clearly described?	Are the main outcomes to be measured clearly described in the Introduction or Methods section?	Are the characteristics of the patients included in the study clearly described?	Are the distributions of principal confounders in each group of subjects to be compared clearly described?	Are the main findings of the study clearly described?	Does the study provide estimates of the random variability in the data for the main outcomes?	Have the characteristics of patients lost to follow-up been described?	Have actual probability values been reported for the main outcomes except where the probability value is <0.001?	Was there potential for recall bias in the ascertainment of the exposure?	Was there potential for differential or non-differential misclassification of the exposure?	Was there potential for observer bias in ascertainment of the outcome?	Was there potential for differential or non-differential misclassification of the outcome?	In trials and cohort studies, do the analyses adjust for different lengths of follow-up of patients, or in case-control studies, is the time period between the intervention and outcome the same for cases and controls?	Were the statistical tests used to assess the main outcomes appropriate?	Were the main outcome measures used accurate (valid and reliable)?	Were the patients in different intervention groups (trials and cohort studies) or were the cases and controls (case-control studies) recruited from the same population?	Were study subjects in different intervention groups (trials and cohort studies) or were the cases and controls (case-control studies) recruited over the same period of time?	Was there adequate adjustment for confounding in the analyses of interest?	Were losses of patients to follow-up taken into account?	Are the study results appropriately interpreted e.g. in terms of the strength of the evidence, its application/ implications, causality?	Did the study have sufficient power to detect a clinically important effect where the probability value for a difference being due to chance is less than 5%?	Total
Pordeus, V.	[101]	No	Yes	Yes	Yes	Yes	No	Yes	No	N/A	Yes	No	No	No	No	N/A	Yes	Yes	N/A	N/A	No	N/A	N/A	N/A	12
Pourahamad, M.	[72]	Yes	Yes	Yes	Yes	Yes	No	Yes	No	N/A	Yes	No	No	No	No	N/A	Yes	Yes	N/A	N/A	No	N/A	Yes	N/A	15
Preiksaitis, JK.	[47]	Yes	Yes	Yes	No	No	No	No	No	N/A	No	No	UTD	No	No	N/A	No	Yes	N/A	N/A	No	N/A	Yes	N/A	9
Puhakka, L.	[28]	No	Yes	Yes	No	No	Yes	Yes	No	N/A	Yes	No	No	No	No	N/A	Yes	Yes	N/A	N/A	No	N/A	N/A	0	12
Qin, HD.	[29]	Yes	Yes	Yes	No	Yes	Partially	Yes	No	N/A	Yes	No	UTD	No	No	N/A	Yes	Yes	N/A	N/A	No	N/A	Yes	5	19
Rahimzadeh, N.	[64]	No	Yes	Yes	No	Yes	No	Yes	No	N/A	Yes	No	No	UTD	No	N/A	Yes	UTD	N/A	N/A	No	N/A	N/A	N/A	10
Ritter, J.	[30]	No	Yes	Yes	No	Yes	Partially	Yes	No	N/A	No	No	No	UTD	UTD	N/A	No	UTD	N/A	N/A	No	N/A	N/A	0	8
Rodriguez, TA.	[51]	No	Yes	Yes	No	Yes	Partially	Yes	No	N/A	No	No	UTD	No	UTD	N/A	No	UTD	N/A	N/A	No	N/A	N/A	0	7
Rubicz, R.	[57]	Yes	No	Yes	Yes	Yes	Partially	Yes	No	N/A	Yes	No	UTD	No	No	N/A	Yes	Yes	N/A	N/A	No	N/A	Yes	0	14
Saghafi-Hendengren, S.	[79]	No	Yes	Yes	No	Yes	Partially	Yes	No	No	No	No	No	UTD	UTD	N/A	Yes	UTD	Yes	Yes	No	No	N/A	N/A	11
Saghafi-Hendengren, S.	[69]	No	Yes	Yes	Yes	Yes	Partially	Yes	No	No	No	No	No	No	No	N/A	Yes	No	Yes	Yes	No	No	N/A	N/A	13
Saghafi-Hendengren, S.	[80]	No	Yes	Yes	Yes	Yes	Partially	Yes	No	No	No	No	No	No	No	N/A	No	No	Yes	Yes	No	No	N/A	N/A	12
Sampaio, MS.	[31]	No	Yes	Yes	No	Yes	Partially	Yes	No	N/A	No	No	No	UTD	UTD	N/A	No	UTD	N/A	N/A	No	N/A	N/A	5	13
Sato, T.	[32]	No	Yes	Yes	Yes	Yes	Partially	Yes	No	N/A	No	No	UTD	No	UTD	N/A	No	UTD	N/A	N/A	No	N/A	N/A	0	8

Lead author	Ref.	Study of risk factors for EBV infection?	Is the study design clearly reported?	Is the hypothesis/ aim/ objective of the study clearly described?	Are the main outcomes to be measured clearly described in the Introduction or Methods section?	Are the characteristics of the patients included in the study clearly described?	Are the distributions of principal confounders in each group of subjects to be compared clearly described?	Are the main findings of the study clearly described?	Does the study provide estimates of the random variability in the data for the main outcomes?	Have the characteristics of patients lost to follow-up been described?	Have actual probability values been reported for the main outcomes except where the probability value is <0.001?	Was there potential for recall bias in the ascertainment of the exposure?	Was there potential for differential or non-differential misclassification of the exposure?	Was there potential for observer bias in ascertainment of the outcome?	Was there potential for differential or non-differential misclassification of the outcome?	In trials and cohort studies, do the analyses adjust for different lengths of follow-up of patients, or in case-control studies, is the time period between the intervention and outcome the same for cases and controls?	Were the statistical tests used to assess the main outcomes appropriate?	Were the main outcome measures used accurate (valid and reliable)?	Were the patients in different intervention groups (trials and cohort studies) or were the cases and controls (case-control studies) recruited from the same population?	Were study subjects in different intervention groups (trials and cohort studies) or were the cases and controls (case-control studies) recruited over the same period of time?	Was there adequate adjustment for confounding in the analyses of interest?	Were losses of patients to follow-up taken into account?	Are the study results appropriately interpreted e.g. in terms of the strength of the evidence, its application/ implications, causality?	Did the study have sufficient power to detect a clinically important effect where the probability value for a difference being due to chance is less than 5%?	Total
Sato, T.	[70]	No	Yes	Yes	No	Yes	Partially	Yes	No	N/A	No	No	UTD	UTD	No	N/A	No	UTD	N/A	N/A	No	N/A	N/A	0	8
Savva, GM.	[58]	No	Yes	Yes	Yes	Yes	Partially	Yes	No	N/A	Yes	No	No	No	No	N/A	Yes	Yes	N/A	N/A	No	N/A	N/A	0	13
Shapira, Y.	[99]	Yes	Yes	Yes	No	Yes	No	Yes	No	N/A	No	No	No	No	No	N/A	Yes	Yes	N/A	N/A	No	N/A	Yes	0	13
Shen, GP.	[59]	Yes	Yes	Yes	Yes	Yes	Partially	Yes	No	N/A	Yes	No	No	No	No	N/A	Yes	Yes	N/A	N/A	No	N/A	Yes	0	16
Simon, KC.	[33]	Yes	Yes	Yes	No	Yes	Partially	Yes	No	No	No	No	No	No	UTD	No	No	UTD	Yes	Yes	No	No	Yes	2	15
Slyker, JA.	[8]	Yes	Yes	Yes	No	Yes	No	Yes	Yes	No	No	No	No	No	No	Yes	Yes	Yes	N/A	N/A	No	Yes	Yes	0	16
Spielmann, G.	[76]	No	Yes	Yes	No	Yes	Partially	Yes	No	N/A	No	No	No	No	No	N/A	No	Yes	N/A	N/A	No	N/A	N/A	N/A	10
Stowe, RP.	[34]	Yes	Yes	Yes	Yes	Yes	Partially	Yes	Yes	N/A	No	No	UTD	No	No	N/A	Yes	Yes	N/A	N/A	No	N/A	Yes	0	15
Stowe, RP.	[82]	Yes	Yes	Yes	No	Yes	No	Yes	No	N/A	No	No	No	No	No	N/A	No	Yes	N/A	N/A	No	N/A	Yes	N/A	12
Sundqvist, E.	[35]	No	Yes	Yes	Yes	Yes	Partially	Yes	No	N/A	Yes	No	No	No	No	N/A	Yes	Yes	N/A	N/A	No	N/A	N/A	0	13
Suntornlohanakul, R	[46]	Yes	Yes	Yes	Yes	Yes	Partially	Yes	Yes	N/A	No	No	No	No	No	N/A	No	Yes	N/A	N/A	No	N/A	Yes	0	15
Tesse, R.	[103]	Yes	Yes	Yes	No	Yes	No	Yes	Yes	N/A	No	No	UTD	No	No	N/A	No	Yes	N/A	N/A	No	N/A	N/A	N/A	10
Thjodleifsson, B.	[77]	No	Yes	No	No	Yes	No	Yes	Yes	N/A	Yes	No	No	No	UTD	N/A	Yes	UTD	N/A	N/A	No	N/A	N/A	N/A	9
Thomas de Montpreville, V.	[61]	No	Yes	Yes	No	Yes	Partially	Yes	No	N/A	No	No	No	No	UTD	N/A	No	UTD	N/A	N/A	No	N/A	N/A	0	8

Lead author	Ref.	Study of risk factors for EBV infection?	Is the study design clearly reported?	Is the hypothesis/ aim/ objective of the study clearly described?	Are the main outcomes to be measured clearly described in the Introduction or Methods section?	Are the characteristics of the patients included in the study clearly described?	Are the distributions of principal confounders in each group of subjects to be compared clearly described?	Are the main findings of the study clearly described?	Does the study provide estimates of the random variability in the data for the main outcomes?	Have the characteristics of patients lost to follow-up been described?	Have actual probability values been reported for the main outcomes except where the probability value is <0.001?	Was there potential for recall bias in the ascertainment of the exposure?	Was there potential for differential or non-differential misclassification of the exposure?	Was there potential for observer bias in ascertainment of the outcome?	Was there potential for differential or non-differential misclassification of the outcome?	In trials and cohort studies, do the analyses adjust for different lengths of follow-up of patients, or in case-control studies, is the time period between the intervention and outcome the same for cases and controls?	Were the statistical tests used to assess the main outcomes appropriate?	Were the main outcome measures used accurate (valid and reliable)?	Were the patients in different intervention groups (trials and cohort studies) or were the cases and controls (case-control studies) recruited from the same population?	Were study subjects in different intervention groups (trials and cohort studies) or were the cases and controls (case-control studies) recruited over the same period of time?	Was there adequate adjustment for confounding in the analyses of interest?	Were losses of patients to follow-up taken into account?	Are the study results appropriately interpreted e.g. in terms of the strength of the evidence, its application/ implications, causality?	Did the study have sufficient power to detect a clinically important effect where the probability value for a difference being due to chance is less than 5%?	Total
van den Heuval, D.	[81]	No	Yes	Yes	Yes	Yes	No	Yes	No	N/A	No	No	No	No	No	N/A	No	Yes	N/A	N/A	No	N/A	N/A	N/A	10
Wang, C.	[60]	No	Yes	Yes	No	Yes	Partially	Yes	No	N/A	No	No	No	No	No	N/A	No	Yes	N/A	N/A	No	N/A	N/A	0	10
Wang, GC.	[75]	No	Yes	Yes	Yes	Yes	Partially	Yes	No	N/A	No	No	UTD	No	No	N/A	Yes	Yes	N/A	N/A	No	N/A	N/A	N/A	11
Wu, JF.	[71]	No	Yes	Yes	No	Yes	Partially	Yes	No	N/A	No	No	No	No	UTD	N/A	No	UTD	N/A	N/A	No	N/A	N/A	0	8
Xiong, G.	[36]	Yes	Yes	Yes	No	Yes	Partially	Yes	No	N/A	No	No	No	No	No	N/A	Yes	Yes	N/A	N/A	No	N/A	Yes	0	14
Xu, FH.	[39]	No	No	Yes	Yes	Yes	Partially	Yes	Yes	N/A	Yes	No	No	No	No	N/A	Yes	Yes	N/A	N/A	No	N/A	Yes	2	17
Yi, B.	[37]	Yes	Yes	Yes	Yes	Yes	Partially	Yes	No	N/A	No	No	UTD	No	No	N/A	No	Yes	N/A	N/A	No	N/A	Yes	0	13
Yu, X.	[66]	Yes	Yes	Yes	Yes	Yes	Yes	Yes	Yes	N/A	Yes	No	No	No	No	Yes	Yes	Yes	Yes	Yes	No	N/A	Partially	N/A	20
Yu, X.	[42]	Yes	Yes	Yes	Yes	Yes	Yes	Yes	Yes	N/A	Yes	No	No	No	No	N/A	Yes	Yes	N/A	N/A	No	N/A	Partially	5	22
Zebrun, AB.	[38]	Yes	Yes	Yes	Yes	No	No	Yes	No	N/A	No	No	No	No	No	N/A	No	Yes	N/A	N/A	No	N/A	Yes	4	16

N/A- not applicable. UTD: unable to determine.
